# Development of a farmworker soil exposure scenario to support the derivation of soil screening levels for per-and polyfluoroalkyl substances (PFAS)

**DOI:** 10.1038/s41370-026-00895-2

**Published:** 2026-04-24

**Authors:** Sara N. Lupolt, Thomas L. Simones, Sara Gillooly, Emily H. Pennoyer, Keeve E. Nachman, Andrew E. Smith

**Affiliations:** 1 Department of Environmental Health and Engineering, Johns Hopkins Bloomberg School of Public Health, Baltimore, MD USA; 2 Risk Sciences and Public Policy Institute, Johns Hopkins Bloomberg School of Public Health, Baltimore, MD USA; 3 Center for a Livable Future, Johns Hopkins Bloomberg School of Public Health, Baltimore, MD USA; 4 Department of Health Policy & Management, Johns Hopkins Bloomberg School of Public Health, Baltimore, MD USA; 5 Maine Centers for Disease Control and Prevention, Augusta, Maine USA

**Keywords:** Exposure, soil contact, occupational health, agriculture, per- and polyfluorinated substances, soil ingestion

## Abstract

**Background:**

Soil screening levels (SSLs) are an important guidance tool for identifying sites where exposure to soil contaminants may pose health risks to people residing or working nearby; however, to date, no soil screening levels or default exposure scenario have been established specifically for farmworkers who have frequent, direct soil contact through their typical occupational tasks and behaviors.

**Objective:**

We developed an exposure scenario for a vegetable farmworker’s soil exposure. We apply this exposure scenario to derive and compare noncarcinogenic-based soil screening levels for four PFAS commonly detected in soil from farm fields with a history of land application of biosolids in Maine.

**Methods:**

We used existing EPA models for incidental soil ingestion and dermal contact, parameterizing model inputs with distributions drawn from the literature to be more representative for farmworkers. We developed an empirical model of the inhalation pathway using literature-reported exposure and time-activity data. We used Monte Carlo simulation to evaluate these models with toxicity values in the EPA Regional Screening Levels database to derive SSLs for the individual and combined pathways and compared these results to soil PFAS levels from a statewide testing program of biosolids-amended farmland.

**Results:**

The 5^th^ percentile values from the distribution of combined pathway SSLs were 26, 7.9, 790, and 0.5 ng/g for PFOS, PFOA, PFNA, and PFDA, respectively. Sensitivity analyses identified the incidental soil ingestion rate as the most influential parameter in deriving a SSL for our farmworker scenario. We found overlap for PFOS, PFOA, and PFDA in the distributions of plausible SSLs for a vegetable farmworker scenario and measured soil levels from a statewide testing program.

**Significance:**

Our comparison of these farmworker SSLs to more routinely evaluated adult exposure scenarios (residential, outdoor worker, construction worker) indicated that these other scenarios may not be protective of farmworkers.

**Impact Statement:**

- The farmworker exposure scenario and PFAS soil screening levels we developed are a novel and necessary public health tool for protecting farmworkers from exposure to contaminants in soils and informing risk management decisions for farms and fields amended with biosolids.

## Introduction

Agricultural workers have frequent, direct contact with soils that may be contaminated with physical, chemical, and biological hazards. They can be exposed to these agents in soils through various pathways, including incidental ingestion, dermal contact, and inhalation of suspended dust. Due to standard agricultural practices like applying biosolids to farmland as fertilizer [1] and the use of pesticides [2], soil and dust may be a reservoir for chemicals and serve as an ongoing source of exposure for farmworkers. Despite this, risks stemming from these exposures are not well characterized, and as a result, there are no regulatory or public health guidelines to protect farmers from soil-related contaminant exposures in agriculture [3]. Much of the published literature on farmworker safety and health has focused on accidents and ergonomic injuries [4], mental health concerns [5], exposures to pesticides [6, 7] and particulates [8, 9]. While attention has been recently directed toward developing exposure scenarios for vulnerable populations and those with greater exposure than the general population [10–12], to our knowledge, there has not been a concerted effort to identify and use pertinent empirical information to develop a farmworker exposure scenario to estimate potential chemical exposure from soils for farmworkers.

The EPA Regional Screening Levels (RSL) for Chemical Contaminants at Superfund Sites calculator is used to develop soil screening levels for several land use scenarios [13]. Establishing an exposure scenario with a set of default exposure parameters is a transparent approach to ensure comparable and consistent assessments, particularly in data-poor contexts. The RSLs are used to identify soil levels of chemicals that may warrant further investigation and action, such as additional testing, remediation, and/or deed restrictions to limit access to contaminated soil [14]. Soil screening levels are based on exposure scenarios which are “a combination of facts, assumptions, and inferences that define a discrete situation where potential exposures may occur” [15]. Within the RSL framework, exposure scenarios are defined for residential, recreational, and occupational exposure scenarios. The occupational exposure scenarios (e.g., outdoor or construction workers) generally rely on the same equations as the residential scenario for incidental soil ingestion and dermal contact pathways but with different inputs and assumptions about incidental soil ingestion rates, duration of exposure, or surface area of skin accessible for soil contact. The EPA’s Preliminary Remediation Goals for Radionuclides at Superfund Sites has equations for a farmworker scenario [16], however it is effectively the residential scenario with a longer exposure duration, and it does not consider the unique aspects of farming activities that may result in increased contact with soil. The EPA default incidental soil ingestion rates used in the RSL framework for residential (adults and children) and occupational worker populations have been designated as low confidence by the EPA [17]. These commonly used exposure scenarios may underestimate soil exposures for farmworkers, given their frequent, direct, and often prolonged contact with soils. For farmworker-specific exposures, there is an emerging body of literature aiming to qualitatively characterize farmworkers’ soil contact [18, 19]. There is a limited body of literature on empirical estimates of soil contact under agricultural scenarios [20, 21] and a single study on modeled estimates of incidental soil ingestion for a high soil exposure scenario [22].

Applying wastewater treatment sludge (or biosolids) is a common agricultural practice in the United States that may pose additional hazards to farmworkers who work in biosolids-amended soils [1]. Biosolids contain various chemicals [23], including per- and polyfluoroalkyl substances (PFAS) [24]. The 2001 EPA National Sewage Sludge Survey estimated land application of between 1375 and 2070 kg/year of 13 measured PFAS [25]. PFAS have been associated with a range of non-cancer and cancer endpoints [26–28]. In 2025, EPA released a draft risk assessment for PFOA and PFOS in sewage sludge that indicated the potential for elevated human health risks to those living on or near impacted farms or primarily relying on food products from these farms [29]. This draft risk assessment considers exposure for a farmworker family but only considers the incidental soil ingestion pathway and uses the same standard EPA default soil ingestion rates as used for residential scenarios [29].

The discovery of high concentrations of PFAS in agricultural soils in Maine from historical land application of biosolids (e.g., 10 to 1000 parts per billion of PFOS) has raised questions about the safety of cultivating foods in these soils and for farmworkers in direct contact with contaminated soils [30–32]. This study describes the development of a farmworker soil exposure scenario and the evaluation of this scenario using Monte Carlo simulations and probabilistic risk assessment methodology to support the derivation of plausible soil screening levels for four PFAS (PFOS, PFOA, PFNA, and PFDA). We compare the derived soil screening levels to PFAS soil levels measured by the Maine Department of Environmental Protection (DEP) and Department of Agriculture, Conservation and Forestry (DACF) on farmland with a history of land-applied biosolids.

## Materials And Methods

### Description of farmworker scenario

The nature of farm work is highly dynamic and depends on the operation’s size (both in land area and personnel employed), crops and growing practices, tools used, and climate, even within the same region. As a result, agricultural exposures can vary significantly. Here, we model a farmworker receptor working on a diversified, small-to-medium-sized operation growing a variety of vegetables primarily during a 9-month growing season. Our scenario assumes a combination of tractor-assisted (with an open cab) and hand-planting, harvesting and weeding. This vegetable farmer scenario was motivated in part by the authors’ experiences responding to PFAS contamination on such farms and the availability of time-activity data to help inform key inputs. We evaluate incidental soil ingestion and dermal soil contact and inhalation of fugitive dust from the soil using EPA RSL equations for the adult residential scenario but replace several default variables with distributions (described below) considered more appropriate for farmworkers. As a departure from default EPA RSL risk assessment methods for soil-related inhalation exposures, we use inputs derived from published estimates of personal exposure monitoring of inhalable dust of farmworkers engaged in work-related activities (e.g., soil preparation and harvesting). This scenario was refined based on empirical data on specific farm tasks and the authors’ direct experience with farms affected by the land application of biosolids in Maine.

### Model overview - probabilistic Monte Carlo simulation

To address variability and uncertainty in the model input parameters, we applied Monte Carlo simulation (MCS) to estimate a distribution of plausible soil screening levels (ng/g) for each exposure pathway individually and for all exposure pathways combined. Unlike traditional risk assessment approaches that use a single deterministic value for each model input parameter, which is usually reflective of a 95th percentile of exposure, MCS uses a distribution of values for model input parameters and, in turn, provides a distribution of model outputs that describe a range of possible risk-based threshold concentrations in soil (ng/g) that account for variability and uncertainty across multiple variables. A protective soil screening level representative of more highly exposed persons (e.g., the 95th percentile) can be interpreted as the 5th percentile of the MCS distribution of soil screening levels.

### Incidental soil ingestion pathway model equations and parameters

The soil screening level for incidental soil ingestion was derived using the following equation [13]:

$$	{{SL}}_{{farmer}{{\_}}{ing}}\left(\frac{{ng}}{g}\right)=\\ 	 \frac{\left({THQ}\times {AT}\left(\frac{{days}}{{yr}}\times {ED}\left({yr}\right)\right)\times {BW}\left({kg}\right)\right)}{\left(\frac{{RBA}}{{RfD}\,\left({ng}/{kg}/{day}\right)}\right)\times {EF}\left(\frac{{days}}{{yr}}\right)\times {ED}\left({yr}\right)\times {{IRS}}_{{farmer}}\left(\frac{{mg}}{{day}}\right)\times \left(\frac{1g}{1000\,{mg}}\right)}$$

The parameterization of each input is described in Table 1. The exposure frequency, exposure duration and incidental soil and dust ingestion rate for a farmworker are the exposure parameters used to derive the soil screening levels (Table 1).

**Table 1:** Input parameters for farmworker soil screening levels.

Parameter	Pathways	Description	Distribution type	Distribution parameters	Reference
**EF**	All	Exposure frequency (days/yr)	Normal	Mean = 256; SD = 17	Maine DEP RAGs 2023 [71]
**ED**	All	Exposure duration (yrs)	-	26	U.S. EPA 2011 [33], Table 16–108
**BW**	All	Body weight (kg)	Normal	Mean = 83.5; SD = 3.3	U.S. EPA 2011 [72], Table 8–3
**AT**	All	Averaging time (days) (365*ED)	-	9490	U.S. EPA 1989 (pg. 6-23) [14]
**THQ**	All	Target hazard quotient	-	1	Maine DEP RAGs 2023 [71]
**RfD**_**PFOS**_	All	Chronic oral reference dose (ng/kg/day)	-	0.1	U.S. EPA RSL Calculator [13]
**RfD**_**PFOA**_	All	Chronic oral reference dose (ng/kg/day)	-	0.03	U.S. EPA RSL Calculator [13]
**RfD**_**PFNA**_	All	Chronic oral reference dose (ng/kg/day)	-	3	U.S. EPA RSL Calculator [13]
**RfD**_**PFDA**_	All	Chronic oral reference dose (ng/kg/day)	-	0.002	U.S. EPA RSL Calculator [13]
**RBA**	Incidental ingestion, inhalation	Relative bioavailability factor (unitless)	Uniform	Min = 0.5; Max = 1	U.S. EPA 2012 [44]
**IRS**_***farmer***_	Incidental ingestion	Incidental soil and dust ingestion rate (mg/day)	Lognormal	GM = 81; GSD = 3.3	Hubbard et al. [22]
**SA**	Dermal	Skin surface area soil (cm^2^/day)	Calculated	^a^	U.S. EPA 2011 [36], Tables 7–2 and 7-12
**AF**_***farmer***_	Dermal	Soil-to-skin adherence factor - farmworker (mg/cm^2^)	Lognormal	Hands: GM = 0.47; GSD = 1.60 Arms: GM = 0.13; GSD = 2.66	Kissel et al. [20]
**ABS**_**d**_	Dermal	Fraction of contaminant absorbed dermally from soil (unitless)	Uniform	Min = 0.01; Max = 0.04	Chen et al. [37]
**GIABS**	Dermal	Fraction of contaminant absorbed in GI tract (unitless)	Uniform	Min = 0.5; Max = 1	U.S. EPA 2004 [38], Exhibit 4-1, section 4.2
**InhRS**_***farmer***_	Inhalation	Inhaled soil particle rate (mg/day)	Calculated	^b^	Nieuwenhuijsen et al. [9] and Lupolt et al. [12]

^a^The skin surface area for hands and arms was calculated in each Monte Carlo simulation using body-part specific formulas from Table 7-7 of the U.S. EPA Exposure Factors Handbook, based on randomly sampled body weight (kg) and height (cm) data from NHANES. Height was modeled as a normal distribution (mean = 167.03 cm; SD = 10).

^b^The inhaled soil particle rate was calculated directly in each iteration from randomly sampled task-specific concentrations and exposure durations (Table S1).

#### Exposure frequency (EF)

We modeled a normal distribution (mean = 256 days per year, SD = 18) to describe the variation in the number of days per year with soil bare and unfrozen using 2001-2017 climatic data representative of the Portland and Bangor, Maine areas. Snow depth and air temperatures were downloaded from the NOAA Applied Climate Information System website (https://scacis.rcc-acis.org/) and an average daily temperature was computed from recorded hourly temperature measurements. The number of days per year with bare, unfrozen ground were calculated by subtracting the number of days per year with both bare (snow depth = 0) and frozen ground (average air temperature < 32° F) from the total number of days per year with bare ground. This distribution does not include EPA’s default value for the residential and construction work scenarios (350 days per year) but is inclusive of the assumed exposure frequency for the outdoor work scenario (225 days per year).

#### Exposure duration (ED)

Exposure duration is the number of years the farmworker works in the impacted field. In noncancer risk assessment, it is also used to compute the averaging time. As a result, ED appears in both the numerator and denominator and ultimately cancels out mathematically. We therefore used a single value for exposure duration of 26 years for the farmworker scenario, equivalent to the duration for the EPA’s residential scenario. This default value is set at the 90th-percentile residential occupancy period of 26 years for males and females, based on a simulation study of residential occupancy periods [33]. We note that this duration is less than the 40-year duration used for a farmworker exposure in the EPA PRGs, but similar to the assumed duration for outdoor workers (25 years) and greater than construction workers (1 year). As a further check on this value’s relevance to farmworkers in Maine, we consulted data from the 2022 United States Department of Agriculture’s Agricultural Census, which found the average number of years for farmers in Maine on any operation ranges between 13.5 and 25.6 years, depending on the type of tenure (i.e., full owner, part owner or tenant) [34].

#### Incidental soil ingestion rate (IRS)

Incidental soil ingestion rate is the amount of soil from impacted fields ingested by the farmworker daily. We modeled incidental soil ingestion as a lognormal distribution (geometric mean (GM) = 81 mg/day; geometric standard deviation (GSD) = 3.3) based on a study that used time-activity and behavioral data to estimate soil ingestion rates with the EPA’s Stochastic Human Exposure and Dose Simulation Soil and Dust (SHEDS-Soil/Dust) model [22]. To develop this estimate, the SHEDS-Soil/Dust model uses EPA’s Consolidated Human Activity Database (CHAD), a compiled database of detailed time-activity and human behavior from 22 separate exposure and time-use studies [35]. The database includes more than 54,000 individual study days of detailed human behavior, each broken down into individual hours and activity types. For a “high soil exposure scenario” Hubbard et al. only sampled those diaries reporting five or more hours outside per day for the spring, summer, and fall seasons, which was intended to represent persons with extensive contact with outdoor soil, including construction, utility workers and farmers or gardeners. Simulations were performed by selecting individuals who spent at least five hours outdoors per day, each with detailed time-activity data. The SHEDS-Soil/Dust model randomly draws from statistical distributions for hand contact rates with soil, soil adherence to hands, fraction of hand surface area contacting the mouth, hand washing that removes soil, and hand-to-mouth frequency.

Our daily estimate is an output of this modeling study and represents the average of the outputs of three seasonal geometric means (spring = 80.5 mg/day; summer = 80.7 mg/day; fall = 81.5 mg/day) for the “high soil” exposure scenario each with a geometric standard deviation of 3.3 (see Hubbard et al. [22] Tables S10–12). This distribution includes EPA-recommended default rates of 100 mg/day for residential adult and outdoor worker scenarios and 330 mg/day for construction worker scenarios [13].

#### Body Weight (BW)

Body weight is the mass (kg) of the farmworker working in the impacted field. We modeled body weight as a normal distribution (mean of 83.5 kg, standard deviation (SD) of 23.7) based on the 2017–2018 NHANES for males and females combined, ages 18–65 years (*n* = 4177).

### Dermal contact pathway model equations and parameters

The soil screening level for dermal contact was derived using the following equation [13]:

$$	{{SL}}_{{farmer}{{\_}}{der}}\left(\frac{{ng}}{g}\right)=\\ 	 \frac{\left({THQ}\times {AT}\left(\frac{{days}}{{yr}}\times {ED}\left({yr}\right)\right)\times {BW}\left({kg}\right)\right)}{\left(\frac{1}{{RfD}\,\left({ng}/{kg}/{day}\right)\times {GIABS}}\right)\times {EF}\left(\frac{{days}}{{yr}}\right)\times {ED}\left({yr}\right)\times {SA}\left(\frac{{{cm}}^{2}}{{day}}\right)\times A{F}_{{farmer}}\left(\frac{{mg}}{{{cm}}^{2}}\right)\times {{ABS}}_{d}\times \left(\frac{1g}{1000\,{mg}}\right)}$$

The parameterization of each input is described in Table 1. The exposure frequency, exposure duration, surface area and the soil-to-skin adherence factor are the exposure parameters used to derive RSLs (Table 1). The assumed exposure frequency and duration are the same as the incidental ingestion pathway.

#### Surface area (SA)

Surface area is the amount of skin assumed exposed (i.e., not covered by clothing or personal protective equipment) on the farmworker while working in the impacted field. In this scenario, we assumed only the hands and forearms were exposed. We modeled the surface area for hands and arms as a function of the randomly sampled distributions of body weight described above, a distribution of height, and using body-part specific formulas from Table 7-7 of the EPA Exposure Factors Handbook [36]. We modeled height as a normal distribution (mean of 167.0 cm, SD of 10.0 cm) based on the 2017-2018 NHANES for males and females combined, ages 18–65 years matched to the same individual data used to compute the body weight distribution (*n* = 4177).

#### Soil-to-skin adherence factor (AF)

Soil-to-skin adherence factor is the amount of soil that sticks to each exposed body part while the farmworker is working in the impacted field. This value is assumed constant throughout the day, and the model does not account for handwashing or periodic removal of soil from the skin surface. We modeled soil-to-skin adherence factors for hands and forearms, separately as lognormal distributions with GM = 0.47, GSD = 1.6 and GM = 0.13, GSD = 2.66, respectively. These distributions were derived using reported GMs and confidence intervals for soil loading on the hands and forearms of farmworkers post-activity in a study examining soil-to-skin adherence for workers under occupational conditions [20]. Two groups of farmers included in this study were presumably vegetable farmers, as they provided produce to a local vegetable market. Sampling was conducted in May and July, representing the spring and summer seasons, with each sample lasting 2 h. These distributions were sampled with their corresponding paired surface area distributions and summed to give a total dermal loading of farmworkers. Lower legs and face were not considered because the published soil-loading estimates for farmworkers were small relative to hands and forearms; furthermore, only two individuals with the one group (July) were noted as wearing shorts rather than pants.

#### Dermal absorption fraction (ABS_d_)

Dermal absorption fraction is the proportion of a chemical absorbed into the body through the skin. We modeled the dermal absorption fraction as a uniform distribution ranging from 0.01 to 0.04, based on the permeable fraction reported from an in vivo dermal absorption study with rats [37]. PFAS in an acetone solution was applied to skin with hair shaved, and PFAS dermal absorption was differentiated into the total amount absorbed following 6-hour contact, the amount retained in skin, and the amount permeable into blood.

#### Gastrointestinal absorption fraction (GIABS)

The gastrointestinal absorption fraction (GIABS) represents the fraction of the contaminant absorbed in the gastrointestinal tract. It is used to modify published oral dose-response metrics (i.e., reference doses and oral slope factors), typically based on an administered dose for dermal exposure estimates of an absorbed dose. When little or no existing literature is available to inform a GIABS, a value of one (i.e., assuming 100%) is typically used [38]. We modeled the GIABS as a uniform distribution (min=0.5; max=1).

### Inhalation pathway model equations and parameters

Resuspended soil dust can be generated by farm machinery, hand tool activity and wind erosion. The soil screening level for inhalation (SL_farmer_inh_) was derived using the following equation [13]:

$$	{{SL}}_{{farmer}{{\_}}{inh}}\left(\frac{{ng}}{g}\right)= \\ 	 \frac{\left({THQ}\times {AT}\left(\frac{{days}}{{yr}}\times {ED}\left({yr}\right)\right)\times {BW}\left({kg}\right)\right)}{\left(\frac{{RBA}}{{RfD}\,\left({ng}/{kg}/{day}\right)}\right)\times {EF}\left(\frac{{days}}{{yr}}\right)\times {ED}\left({yr}\right)\times {{InhRS}}_{{farmer}}\left(\frac{{mg}}{{day}}\right)\times \left(\frac{1g}{1000\,{mg}}\right)}$$

The parameterization of each input is described in Table 1. The exposure frequency and exposure duration are the same as the incidental ingestion and dermal contact pathways (Table 1).

#### Inhaled soil dust rate (InhRS_farmer_)

The inhaled soil dust rate is the daily mass of resuspended soil dust particles from the impacted field (mg/day) inhaled by the farmworker. The nature of agricultural work adds increased complexity to characterizing inhalation exposures to resuspended soil dust in two notable ways: 1) farmers may engage in activities that increase the amount of soil particles in the air, and 2) farmers may have different inhalation rates for different activities, depending on their level of exertion. We used two farmworker-specific datasets to derive an agriculture-specific data inhalation rate of soil dust [9, 12]. We derived an hourly inhalation rate of soil particles from personal monitoring data on inhalable dust concentration collected during farming activities from a published study, typical inhalation rates for moderate activity and task-specific time-activity information for growers using the following equation:

$${{InhRS}}_{{Farmer}}={\sum}_{i=1}^{n}{C}_{{air},i}\left(\frac{{mg}}{{m}^{3}}\right)\times {{{\rm{InhRH}}}}\left(\frac{{m}^{3}}{{hr}}\right)\times \frac{{{ED}}_{i}\,\left({hrs}\right)}{{EF}}\,$$

Where C_air_ is the distribution of inhalable dust concentration (mg/m^3^) measured for each of five farm-related tasks [9] *InhRH* is a short-term moderate-intensity inhalation rate (m^3^/hr) for adults, and ED_i_ is the number of hours spent on a farm-related task *i* and EF is the number of days per growing season as previously described (Table S1).

We reviewed published literature to identify studies reporting inhalable and/or respirable dust levels measured or calculated from personal monitoring of farmers while engaging in farm work. Nineteen article abstracts were reviewed, with five articles retained for full review of inhalable [39–41] (*n* = 3) and both inhalable and respirable [9, 42] (*n* = 2) dust measurement results. These five articles included measurements by farm-related task (e.g., ground preparation, planting, harvesting) or by type of farmer without specifying tasks (e.g., grain farmer, vegetable crop farmer, cabbed and uncabbed tractor drivers). There was variability between studies in sampling duration (e.g., task-specific vs. workday) and sampling equipment used, though three studies used the Institute of Occupational Medicine (IOM) inhalable dust sampler at two liters per minute to collect inhalable dust, so they are comparable in terms of sampling equipment. It was generally noted that exposure assessment of farmworkers is difficult for many reasons, including changes in planned work, weather-related adjustments to the work schedule, the varied and cyclical nature of agricultural work, the diversity of farms and fields, and behavioral differences between farmers, among others. We selected Nieuwenhuijsen et al. [9], as it was the only study to conduct year-round sampling of farm-related activities in both wet and dry seasons and thus provides a better representation of farm-related exposures under different environmental conditions. The measured inhalable dust levels across seven distinct farming tasks ranged from 0.7 to 11.74 mg/m^3^ and 2.51 to 51.81 mg/m^3^ for average and 95th percentile estimates, respectively, with some tasks further differentiated by the commodity produced and use of mechanized equipment (with both open and closed cab). We modeled the concentrations of C_air_ for five tasks (i.e., ground preparation, seeding/transplanting, mechanical weeding, and harvesting vegetables) most likely to be conducted by Maine farmers as lognormal distributions using the reported GM and GSD (Table S1).

For an inhalation rate, we modeled a normal distribution (mean=1.8 m^3^/hr, SD = 0.49) representing short-term moderate-intensity activities for adults [43] (Table 6–2 in the Exposure Factors Handbook).

The exposure duration (in hours spent on each task) was informed by a time-activity soil contact activity questionnaire administered to 38 agricultural workers in Maryland between April 2020 and March 2022 [12]. Briefly, for each of the four seasons, farmworkers provided estimates of time spent (hours per month) engaged in six tasks over the previous 30-day period. These 30-day estimates for five tasks from the Nieuwenhuijsen study for which inhalable dust measurements were available were multiplied by 3 to estimate hours spent across the traditional 3-month season. Seasonal estimates for the spring, summer, and fall seasons were summed to estimate time spent on these activities, approximately equal to a typical growing season or exposure frequency (i.e., 256 days). We calculated the GMs and GSDs in Excel for the task-specific exposure durations reported by the farmers (*n* = 23) who completed questionnaires in all three seasons. We modeled a lognormal distribution for bed preparation, seeding, transplanting, and weeding tasks, and a uniform distribution, using maximum and minimum values (min=18.75 h, max=607.5 h) for the harvesting task. Because Nieuwenhuijsen et al. reported inhalable dust concentrations for hand and mechanized harvesting separately, we assumed each randomly sampled exposure duration for harvesting was divided evenly between hand and mechanized harvesting.

### PFAS-specific toxicological considerations

For the incidental soil ingestion and inhalation pathways, the relative bioavailability factor (RBA) accounts for differences in bioavailability of the medium (e.g., soil) and the medium associated with the toxicity value [44]. Published data on the bioavailability of several PFAS following oral exposure in animal bioassays are available. These data indicate that several PFAS are effectively absorbed, typically >95%, from the gastrointestinal tract in animal oral dosing studies when the exposure medium is water [27]. Similarly, several studies have shown negligible reductions in the bioavailability of PFOA or PFOS in feed in chickens [45, 46] and dairy cows [47, 48]. However, there is limited data on the absorption of various PFAS in soil. Due to the absence of robust data on the bioavailability of PFAS from soil, we modeled RBA as a uniform distribution (min=0.5; max=1).

For the chronic oral noncancer toxicity values, we used reference doses (RfDs) provided in the EPA RSL database as of November 2024 for four individual PFAS as follows: PFOS, PFOA, PFNA, PFDA at 0.1, 0.03, 3, 0.002 ng/kg/day, respectively (Table S2). These toxicity values reflect those currently included in the EPA RSL Calculator selected by EPA according to EPA hierarchy for selecting toxicity information [13]. These four PFAS were selected a priori due to their high detection prevalence across testing efforts in Maine to assess contamination in agricultural lands with a history of biosolids application. In the absence of inhalation toxicity values, inhalable soil dust levels were treated as an ingested dose to which an oral RfD was applied.

We set a target hazard quotient (THQ) value of one for each pathway individually for each PFAS-specific soil screening level we derived (Table 1). For all pathways, we assumed an averaging time of 9,490 days (equal to 365 days of potential exposure per year over the exposure duration of 26 years) (Table 1). The averaging time is the total amount of time encompassing the exposure scenario and represents the maximum number of days over which the exposure is divided.

### Combined screening level for all pathways

For each PFAS, we summed the individual ingestion, dermal, and inhalation pathway equations into a single algebraic expression (SI). This produced a single combined soil screening level that accounts for the cumulative contribution from all pathways and enabled MCS using consistent sample draws (e.g., a single bodyweight draw applied across all pathways) [13].

### Cancer risk calculation

To examine whether a cancer-based SSL could drive a lower SSL than noncancer effects, we estimated the incremental lifetime cancer risk for PFOA and PFOS, the only PFAS with cancer slope factors (CSFs) included in the EPA RSL database (29,300 and 39.5 per mg/kg/day, respectively). We used the exposure level corresponding to the 5th percentile noncancer SSL, which represents the 95th percentile exposed farmworker and, using a THQ of 1, is equal to the RfD. Cancer risk was then calculated by multiplying the RfD for PFOA and PFOS by their respective CSF and using a lifetime averaging time of 70 years (SI).

### Statistical analyses

Monte Carlo simulation analyses were performed in R (version 2025.09.2 + 418). We assigned 10,000 simulations to each parameter and limited draws from parameter distributions to ± 2 standard deviations to avoid extreme values [49, 50]. Descriptive statistical analyses of MCS outputs and violin plots (a combination of a box plot and a kernel density plot) were also performed in R. As a sensitivity analysis, we investigated the relative importance of individual input parameters on the SSL for each exposure pathway and for all pathways combined. Sensitivity of the model-predicted SSLs was assessed using the *tornado* package in R, which estimates the range of SSLs expected when each input parameter is independently shifted to its 5 and 95th percentile while all other parameters are held at their mean values. The resulting tornado plots illustrate the SSL's sensitivity to varying inputs, with longer bars indicating greater sensitivity.

### PFAS soil distributions

Maine’s *Act to Investigate Perfluoroalkyl Substance Contamination of Land and Ground Water* (Public Law 2021 Chapter 478) requires the DEP to systematically collect soil samples from all licensed sludge and septage land application sites in Maine for PFAS analysis. In addition, the Maine Department of Agriculture, Conservation and Forestry (DACF) has assisted the DEP in sampling fields with a history of biosolids application at active farm sites where DACF is involved. Briefly, the boundaries of the sample area are designated within a licensed biosolids-applied field based on the field’s shape and size. If the field is larger than 10 acres DEP typically samples a 10-acre subarea of the field. DEP collects a 10-part composite soil sample for each of the designated areas. The DACF standard field soil collection practice is to sample the entire field with a 30-part composite soil sample, with the sampler attempting to evenly space the composite samples throughout the field in a grid-like fashion. Both agencies collect individual soil samples with a 2.5 cm diameter push probe at a 15 to 20 cm depth. All samples are collected following DEP Standard Operating Procedures for soil sampling and sampling for PFAS [51]. PFAS were measured in soil using commercial laboratories as described elsewhere [30]. To summarize statewide soil PFAS concentrations in soil on biosolid-applied fields, data from the DEP Environmental and Geographical Analysis Database (EGAD) and the PFAS Investigation Map were accessed. Fields not designated as sludge utilization sites were excluded. Percentiles of PFAS soil concentrations were calculated for 875 fields with measured PFAS concentrations. The soil PFAS concentration results are representative of statewide testing up to October 2025.

## Results

### PFAS soil screening levels

**Fig. 1:**
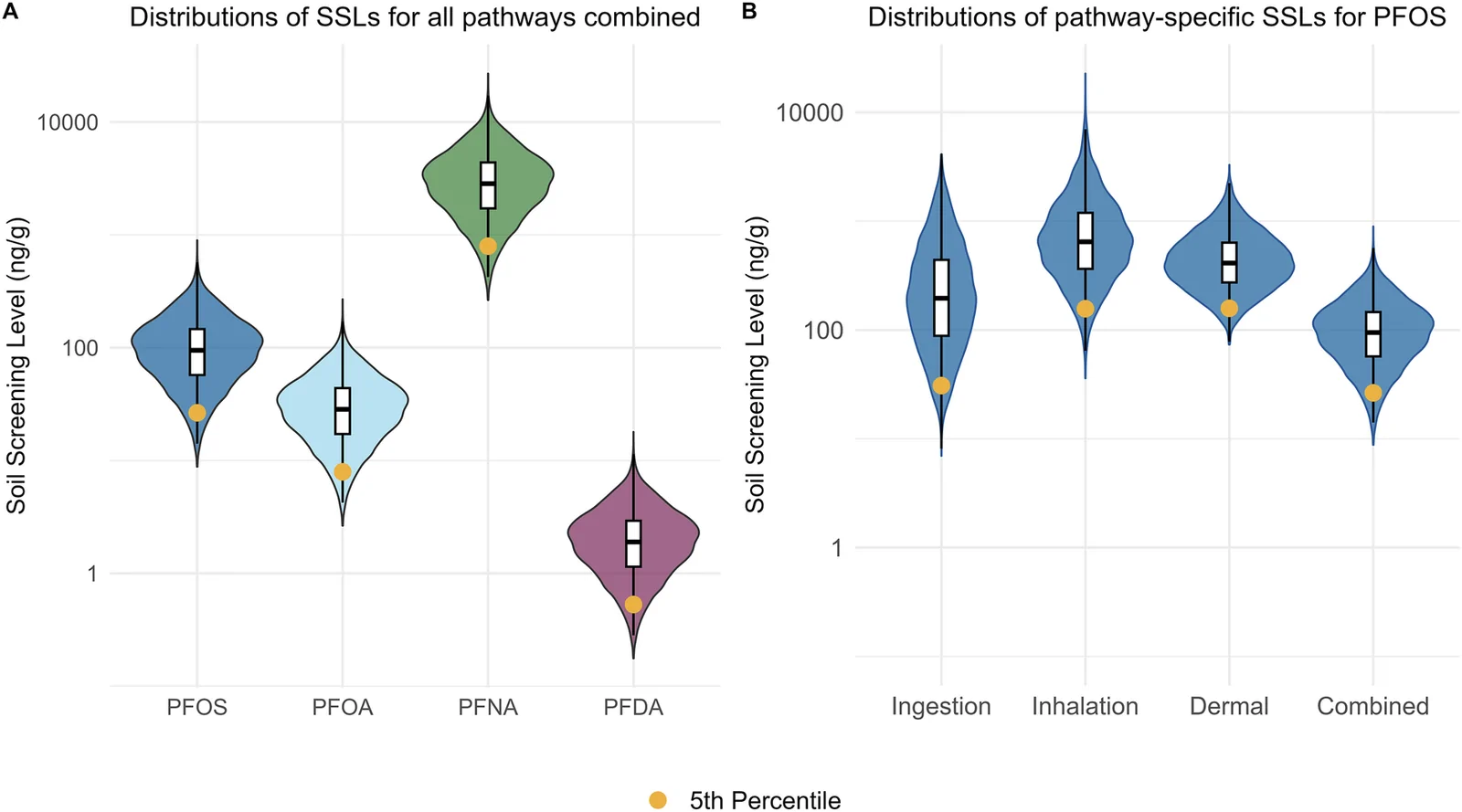
Individual and combined Soil Screening Levels (SSLs) for a farmworker exposure scenario in Maine. **A** Distributions of farmworker SSLs for four PFAS across all exposure pathways combined. **B** Pathway-specific distributions of SSLs for perfluorooctanesulfonic acid (PFOS). Violinplots show the probability density of values, with boxplots indicating the median, interquartile range, whiskers (1.5 x IQR), and the 5th percentile.

Using MCS, we derived distributions of farmworker scenario soil screening levels for each PFAS for the ingestion, dermal and inhalation pathways, as well as a combined soil screening level for all pathways. Selected percentiles of these distributions are presented in Table 2 and the full distributions are shown as violin plots in Fig. 1. As it is a common risk assessment practice to establish soil screening levels protective of highly exposed individuals, we highlight the 5th percentile soil screening level as it is protective of the 95th percentile exposed individual. The 5th percentile soil screening levels for combined pathways ranged from 0.5 ng/g (PFDA) to 790 ng/g (PFNA). The incidental soil ingestion pathway resulted in lower SSLs than the other pathways and was also more variable based on inspection of percentiles, interquartile range, and the shape of the distributions in the violin plots. The dermal contact and inhalation pathways had similar distributions, although the inhalation pathway was more variable and had a lower 5th percentile SSL. The incidental soil ingestion pathway was the driver of the combined SSL at the 5th percentile, but both dermal and inhalation pathways were influential on the combined SSL at the 50th percentile (Table 2). Because the only PFAS-specific input in our calculations is the toxicity value, the resulting MCS shapes of the distributions are identical across pathways shifted by different toxicity values (Fig. 1).

**Table 2:** Summary of pathway-specific noncarcinogenic farmworker soil screening levels (ng/g) for four PFAS most frequently detected in Maine.

	Soil screening level (ng/g)^a^			
Pathway	5th percentile	25th percentile	50th percentile	75th percentile
**PFOS**				
Incidental Soil Ingestion	31	88	200	440
Inhalation	160	360	650	1200
Dermal Contact	160	270	410	630
Combined	26	57	95	150
**PFOA**				
Incidental Soil Ingestion	9.2	27	59	130
Inhalation	47	110	190	360
Dermal Contact	47	82	120	190
Combined	7.9	17	28	44
**PFNA**				
Incidental Soil Ingestion	920	2700	5900	13000
Inhalation	4700	11000	19000	36000
Dermal Contact	4700	8200	12000	19000
Combined	790	1700	2800	4400
**PFDA**				
Incidental Soil Ingestion	0.62	1.8	3.9	8.8
Inhalation	3.1	7.3	13	24
Dermal Contact	3.2	5.5	8.2	13
Combined	0.53	1.1	1.9	2.9

^a^All SSLs were rounded to 2 significant figures.

For each PFAS, the farmworker scenario produced 5th percentile noncancer combined pathway SSLs that were lower than those derived using the EPA RSL calculator for the residential adult and outdoor worker scenarios and similar to the construction worker scenario (Table 3). The construction worker scenario assumes exposure at a given site is limited to a single year, which would impact the derivation of a cancer SSL. A SSL for the residential child scenario soil has the lowest SSL but would only be applicable in the farming scenario to the land in proximity to the farmer’s residence, rather than more remote fields.

**Table 3:** Comparison between the 5th percentile of overall noncarcinogenic Farmworker soil screening levels (ng/g) and Residential, Outdoor Worker, and Construction Worker scenarios for four PFAS frequently detected in Maine.

PFAS	5th Percentile Farmworker SSL	^a^Residential_child_ SSL	Residential_adult_ SSL	Outdoor Worker SSL	Construction Worker SSL
Perfluorooctanesulfonic acid (PFOS)	26	8.6	80	112	25
Perfluorooctanoic acid (PFOA)	7.9	2.6	24	34	7.6
Perfluorononanoic acid (PFNA)	790	259	2410	3360	762
Perfluorodecanoic acid (PFDA)	0.5	0.2	1.6	2.2	0.5

^a^The Residential, Outdoor Worker, and Construction Worker soil screening levels are derived using the EPA RSLcalculator with Maine DEP Remedial Action Guideline (RAGs) specific exposure parameters and the current as of Nov 2024 noncancer toxicity values in the RSL database [28]. Because exposure frequency was based on Maine-specific climate data, SSLs in regions with fewer days of snow-covered soils would be lower. For the inhalation pathway in the EPA RSL calculator, oral RfDs were adjusted to RfCs based on route-to-route extrapolation using body weight (80 kg) and inhalation rate (20 m^3^/day) [73, 74].

Using the available EPA cancer slope factors, we estimated that our 5th percentile noncancer SSL for PFOA and PFOS correspond to incremental lifetime cancer risks of approximately 3.3 × 10^-4^ and 1.5 × 10^-6^, respectively.

### Sensitivity analyses

In sensitivity analyses, the combined SSL was strongly influenced by the incidental soil ingestion rate parameter (Fig. S4). The PFOS SSL varied from 30 to 262 ng/g when the soil ingestion rate varied between its 95 and 5th percentile values, respectively. The next four most influential parameters were body weight, relative bioavailability, fraction absorbed dermally, and particulate matter from mechanical harvesting. The variability in all other parameters had a limited impact on the combined pathway SSL. These parameters were more influential when examined in sensitivity analyses of the individual pathway SSLs (Figs. S1-4). For the dermal pathway, the fraction of soil adhering to skin that was absorbed and soil loading onto hands were the most influential parameters. For the inhalation pathway, the concentration of particulate matter in air while harvesting vegetables with a tractor and the time spent weeding and harvesting vegetables over the growing season were the most influential parameters on the SSL [52].

### Comparison of soil screening levels to measured PFAS at sites with a history of biosolids land-application

**Fig. 2:**
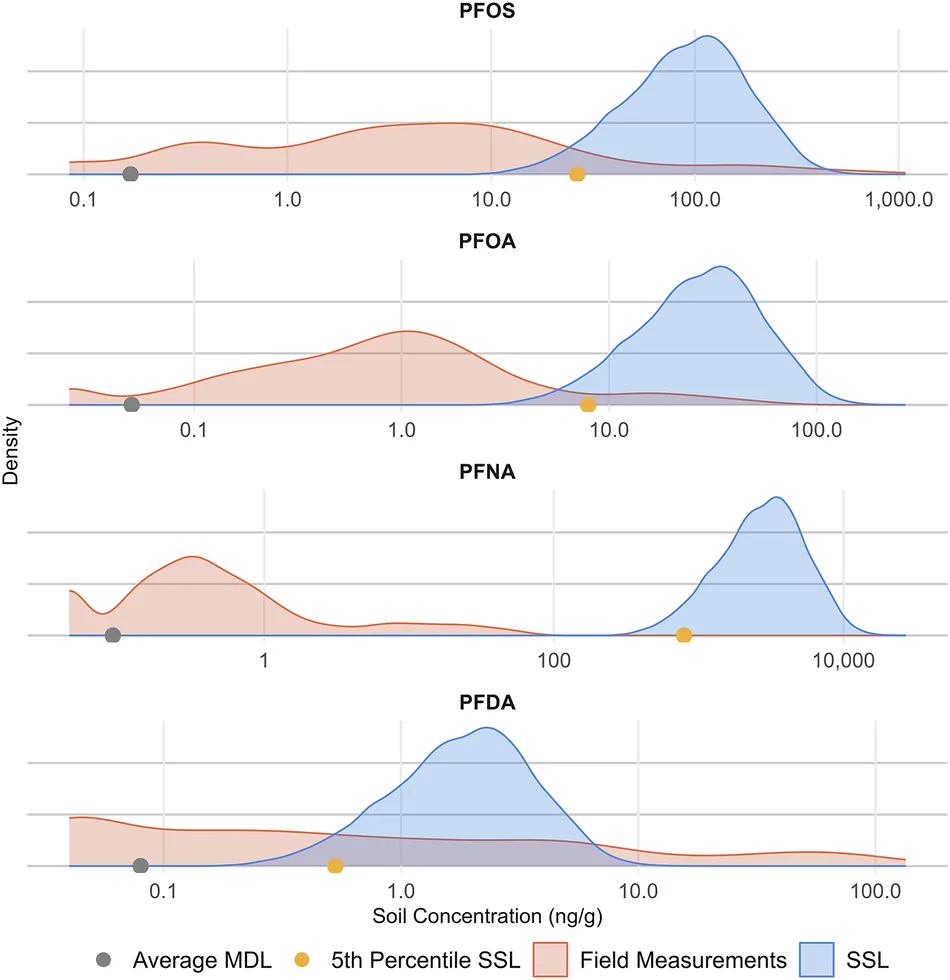
Illustration of the distribution of soil screening levels for all pathways combined compared to field measurements of PFAS from farm fields in Maine with a history of biosolids application (*n* = 875). Field measurements below the method detection limit (MDL) were replaced with ½ of the mean MDL. The mean MDLs were 0.05 (PFOA), 0.17 (PFOS), 0.08 (PFDA), and 0.09 (PFNA).

As of October 2025, 875 fields had been tested in Maine in response to PL 2021 Chapter 478. PFOS and PFOA were detected in nearly all tested fields, and PFNA and PFDA were detected in over three-quarters of tested fields (Table 4). Figure 2 illustrates the overlap of the distributions for measured soil levels and SSLs. For PFOS, 12.9% of the tested fields had soil concentrations exceeding the 5th percentile farmworker SSL of 26 ng/g. Field soil concentrations for PFOA, PFNA, and PFDA exceeded the 5th percentile SSLs in 7.9%, 0%, and 44.7% of the tested fields, respectively. PFOA, while detected in soil at a similar frequency as PFOS, was typically found at lower concentrations, and accordingly, fewer fields exceed a 5th percentile farmworker SSL of 7.9 ng/g. The maximum PFNA concentration detected in a biosolid spread field was 62 ng/g, well under a 5th percentile SSL of 790 ng/g. The lowest farmworker soil screening level of 0.5 ng/g for PFDA is exceeded in a 43.8% of the tested fields. This PFDA soil screening level is closer to background concentrations measured at unimpacted, non-urban locations in Maine, although it is still above the statistical upper limits for PFDA in unimpacted soils [31].

**Table 4:** PFOS, PFOA, PFNA and PFDA soil concentrations (ng/g) in 875 Maine fields with licensed biosolid applications.

PFAS	5th Percentile Farmworker SSL	Percent detection	25th percentile	50th percentile	75th percentile	90th percentile	95th percentile	Max
Perfluorooctanesulfonic acid (PFOS)	26	95%	0.85	3.8	11.8	43.6	155	1080
Perfluorooctanoic acid (PFOA)	7.9	95%	0.28	0.85	1.9	4.9	14.4	61.4
Perfluorononanoic acid (PFNA)	790	86%	0.16	0.35	0.9	6.7	17.5	61.6
Perfluorodecanoic acid (PFDA)	0.5	75%	0.07	0.37	3.4	24.1	58.2	134

## Discussion

Our work is motivated by the discovery of contaminated farm soils in Maine, with PFOS levels ranging from <10 to >1000 ng/g due to the historical land application of biosolids [38, 39]. To our knowledge, this study represents the first effort to establish an exposure scenario to describe and consider agricultural workers’ contact with soil in the derivation of public health guidelines for soil contaminants. For the incidental soil ingestion and dermal contact exposure pathways, we used existing EPA soil screening-level models and adjusted several key parameters to better reflect potential farmworker exposures. For the inhalation pathway, we used a more empirical approach than EPA models for fugitive dust, based on personal exposure monitoring of inhalable dust among farmworkers engaged in a range of mechanized and hand activities, combined with time-activity data. We evaluated these models using MCS methods that treat input parameters as variables with specified probability distributions to generate a distribution of SSLs. As it is standard risk assessment practice to set SSLs that protect highly exposed persons, we compared the 5th-percentile SSLs (protective of the 95th-percentile exposed person) with existing deterministic SSLs. Our farmworker soil screening levels were lower than EPA RSL-based soil screening levels for adult residential and outdoor worker scenarios and were similar to those for construction worker scenarios. We compared our farmworker soil screening levels to Maine statewide soil data from testing of 875 fields with a history of biosolids land application. PFOS and PFDA had the highest percent exceedances of the 5th percentile SSL of measured soil levels at 13% and 44% of tested fields, respectively. Our results indicate a need to better understand and characterize farmworker soil exposure pathways to ensure that models are appropriately protective while remaining reasonably accurate in estimating potential PFAS exposure.

Sensitivity analyses indicated incidental soil ingestion rate was the most influential input for variation in the SSL. To our knowledge, there are no published empirical estimates of incidental soil ingestion by farmworkers. We used modeled estimates of a high-soil-exposure group derived from the SHEDS-Soil/Dust model [22]. This modeling approach has previously been applied to generate incidental soil ingestion estimates for children and has been reported to yield central estimates consistent with empirical observations from trace-element mass-balance studies [53]. It is unknown how representative these modeled estimates of a high-soil-exposure group are of farmworkers. One of the key model inputs (soil-to-skin adherence) was informed by empirical data from farmworkers, but most other inputs were not (e.g., hand-to-mouth frequency, hand contact rate with soil, washing of hands). Time-activity information was based on EPA CHAD daily diaries [35] and limited to persons reporting five or more hours outside. To our knowledge, few of the roughly 200 to 400 daily activity diaries that meet the time-outside requirements are representative of farmworkers. The distribution we used has a 95th percentile soil ingestion rate of 475 mg/day for soil only (i.e., does not include indoor dust exposure). This is higher than the recommended EPA default for developing soil screening levels for adult residential and outdoor worker scenarios (100 mg/day) and for construction workers (330 mg/day). EPA has stated that its confidence in estimates of soil ingestion rates for both adults and children is low, highlighting the limitations of both measured and modeled estimates [17]. Two trace element mass balance studies of Canadian Aboriginal populations engaged in rural wilderness lifestyles (hunting, fishing, camping, gathering) reported 90 or 95th percentile soil ingestion rates ranging from 110 to 361 mg/day [54, 55]. The authors considered these populations representative of medium soil contact activities. EPA opined that these two studies may be appropriate for high-soil-contact scenarios [17]. Given the paucity of published estimates of incidental soil ingestion rates for adults, research is needed to generate farmworker-specific time-activity data to inform estimates of incidental soil ingestion. Methodologies to accomplish this include trace-element mass-balance studies and documentation of farmworker time-activity patterns that can inform inputs to the SHEDS-Soil/Dust model. In addition, work to identify new candidate tracers that may serve as better indicators of soil ingestion is needed to advance tracer approaches that can be used to validate models (e.g., SHEDS-Soil/Dust) and improve estimation of soil and dust ingestion [41]. The Agricultural Health Study may present another opportunity to collect much-needed information about potential soil exposure among farmworkers.

For the dermal pathway, sensitivity analyses indicated that the most influential parameters were the fraction of contaminant absorbed from soil adhering to skin and the loading of soil onto hands and forearms. Studies evaluating the dermal bioavailability of PFAS from aqueous solutions found absorption was chain-length dependent, with absorption decreasing with increasing chain length [37, 56]. In an in vivo rat model, the absorbed fractions penetrating to serum for PFOA, PFNA, PFDA, and PFOS were all <4% [37]. In a human skin equivalent in vitro model, absorbed fractions for PFOA, PFNA, PFDA, and PFOS were 13.5%, 0.9%, 0.1%, and 1.3%, respectively [56, 57]. A separate in vitro human skin study evaluating aqueous dermal absorption for only PFOA reported a higher absorption fraction of 24% penetrating full-thickness skin [58]. However, a study involving the application of sunscreen with added isotope-labeled PFOA to a single human volunteer calculated an absorbed dose of 1.6% from a non-aqueous application [59]. To date, no studies have evaluated dermal absorption from PFAS in contaminated soil, and it is unclear what fraction of soil PFAS is bioaccessible and would be available to permeate the skin and subsequently absorb into blood. It has been reported that more than 60% of PFOA and PFOS present in spiked household dust was bioaccessible in vitro in a 1:1 synthetic sweat: sebum solution and would be available for dermal absorption; the presence of sunscreen increased bioaccessibility [57, 58]. The limited available empirical data support the use of an absorbed fraction for these longer-chain PFAS that is lower than the default EPA value of 0.1 (10%), though this default value has been recently used to assess dermal exposure to PFAS for recreational shellfish harvesters [60]. For soil adherence factors, we used hand- and forearm-specific soil adherence factors that were derived from empirical studies of farmworkers; these studies are of small sample size, are not linked to specific activities, and few of the individuals were wearing shorts that might influence loadings onto the lower legs that we did not consider in our analysis [57, 58].

Inhalation is the route most informed by farmworker-specific empirical observations, including personal exposure monitoring of inhaled dust levels during farm tasks and time-activity data. It differs from how the EPA RSL models inhalation for outdoor or construction workers. Sensitivity analyses identified time spent harvesting and particulate matter generated during mechanical harvesting as the two most variable and influential inputs. For the inhalation pathway, we made some conservative assumptions, including that all measured inhalable dust consisted of resuspended soil particles and all inhaled dust was internalized and contributed to exposure. A previous study suggested that only 50% of inhaled dust of larger particle sizes reaches the intestinal tract [52]. Though we did apply the same RBA term used with incidental soil ingestion to allow for less absorption of PFAS from soil relative to what is expected absorption associated with the studies used to derive the RfDs. We conservatively modeled a vegetable farmworker scenario with appreciable use of open cab tractors for tasks including bed preparation, planting, weeding, and harvesting, which are associated with higher inhalable dust levels than either performing such activities in an enclosed cab or by hand [9]. Better time-activity data for farmworkers and more representative personal exposure monitoring of inhalable (and respirable) dust levels for farmworkers from the Northeast are needed to strengthen these modeling inputs.

Standard risk assessment practice is to derive SSLs for both noncancer and cancer endpoints, when possible, and to use the lower of the two for regulatory purposes. Using the available EPA cancer slope factors (SI), we estimated that our 5th percentile noncancer SSL for PFOS and PFOA correspond to incremental lifetime cancer risks of approximately 1 × 10^-6^ and 3 × 10^-4^, respectively. Whether these cancer risk levels would result in a lower SSL than the noncancer SSL would depend on the chosen acceptable cancer risk level. In promulgating its final Part 503 biosolids rule, EPA made a policy decision to regulate the risk for all biosolids use practices, including risks to farmers and farmworkers, at a cancer risk level of 1 × 10^-4^ [61].

In this study, we compared the distribution of farmworker-specific soil screening levels with that of measurements of four PFAS commonly detected in agricultural soils in Maine with a history of biosolids land application. This was possible because of legally mandated, proactive, and ongoing testing and reporting of PFAS soil concentrations by the Maine DEP. To our knowledge, these data are the first statewide surveillance of PFAS soil levels in farm fields with a history of land application. We found a relatively small fraction of fields had PFOS and PFOA concentrations that exceeded a conservative 5th percentile farmworker SSL. All measured soil levels of PFNA were below the 5th percentile SSL. Approximately 44% of tested fields had soil levels above the PFDA SSL, which is based on the lowest RfD out of the four PFAS examined. The overlap of these two distributions is sensitive to the choice of toxicity values. There are substantial differences in the PFAS RfDs and CSFs derived in recent years by both federal [62, 63], state [64] and international agencies [65]. For example, there is a 29-fold difference in PFOA RfDs and a 10-fold difference in CSFs published by EPA and CA OEHHA in 2024. Future work should evaluate whether the estimated exposures for this farmworker scenario are sufficient to result in a demonstrable increase in blood serum concentrations, given known background serum levels, to inform the need for any measured interventions to protect farmworkers.

We chose to model our farmworker exposure scenario as a small to medium-sized vegetable farm with the use of open-cab tractors rather than tractors with closed cabins. Better characterization of the scope and range of tasks involved in food production (e.g., growing and harvesting crops vs. animal husbandry) and the level of mechanized equipment used (closed and open cab tractors, walk behind tractors) are needed to understand what type or modalities of agriculture are best represented by this exposure scenario. For example, better characterization of farmworkers’ time-activity patterns and frequency of specific agricultural practices would be a valuable tool for gross-tuning of key tasks and practices that may drive exposure. We also note that farmland application of biosolids is another important and relevant exposure scenario with different considerations that is worthy of further investigation.

We focused on PFOS, PFOA, PFDA, and PFNA in this effort because soil measurement data and high-quality toxicity values were available. Other PFAS, including precursors, can also be available for exposure and co-occur and contribute to risk, even after particular compounds are no longer being used [66–68]. The farmworker scenario we developed could be used to derive soil screening levels for additional PFAS and other chemicals present in agricultural soils. This is overdue, given that many agricultural sector workers may be concurrently exposed to other chemical and non-chemical stressors, which may render them more vulnerable to health effects resulting from soil exposure [43]. Further, our approach examined each of the four PFAS independently and did not explore potential interactions among them, additive or otherwise. Recognizing the call to address chemical classes in risk management [69, 70], there is a need to develop methods for setting soil screening levels that account for common mixtures within an exposure medium.

The EPA could also consider the three soil exposure pathways in our farmworker scenario in pesticide registration risk assessments, particularly since some pesticides (including those containing PFAS) may accumulate in soil. Our farmworker scenario could also better inform risk management decisions regarding ongoing exposures after biosolids land application. The EPA’s 2025 draft sewage sludge risk assessment for PFOA and PFOS evaluated soil contact scenarios that considered only the incidental soil ingestion pathway for a family working and residing on a farm where biosolids were land-applied, omitting exposures via dermal contact and inhalation. Thus, these risk estimates may underestimate the risks among farmworkers with ongoing, direct contact with soils amended with biosolids via all pathways after land application. Our farmworker exposure scenario provides an important first step toward developing a farmworker exposure scenario for use in risk assessment.

## Supplementary information

Supplemental Material

## Data Availability

R code for conducting the probabilistic Monte Carlo simulations will be made available upon reasonable request from the authors. PFAS soil measurement data are available from the Maine Department of Environmental Protection EGAD (Environmental and Geographic Analysis Database), https://www.maine.gov/dep/maps-data/egad/.
