# Supplementary material for: Development of a farmworker soil exposure scenario to support the derivation of soil screening levels for per-and polyfluoroalkyl substances (PFAS)
Source: J Expo Sci Environ Epidemiol. 2026 Apr 24;36(6):1160–70. doi: 10.1038/s41370-026-00895-2 (PMC13727675; doi:10.1038/s41370-026-00895-2)
Supplement: Supplementary file 1 [file 41370_2026_895_MOESM1_ESM.docx]

**Supplemental Material**

**Development of a farmworker soil exposure scenario to support the derivation of soil screening levels for per-and polyfluoroalkyl substances (PFAS)**

Sara N. Lupolt, Thomas L. Simones, Sara Gillooly, Emily H. Pennoyer, Keeve E. Nachman, Andrew E. Smith

**Table of Contents**

**Supplemental tables**

**Table S1.** Summary of inputs for the derivation of inhaled soil particulate rate (InhRS_farmer_)

**Table S2.** Summary of the EPA RSL calculator selected reference doses and toxicity endpoint basis for PFOS, PFOA, PFNA, and PFDA

**Figure S1.** Tornado plot for the ingestion pathway soil screening level

**Figure S2.** Tornado plot for the dermal pathway soil screening level

**Figure S3.** Tornado plot for the inhalation pathway soil screening level

**Figure S4.** Tornado plot for the combined pathway soil screening level

**Equation S1.** Combined soil screening level equation for farmers

**Supplemental information.** Cancer risk estimates for PFOS and PFOA

**Table S1. Summary of inputs for the derivation of inhaled soil particulate rate (InhRS_farmer_)**

| Parameter | Description | Distribution type | Distribution parameters | Reference |
| --- | --- | --- | --- | --- |
| C_air-BP_ | Particulate matter concentration for bed preparation (mg/m^3^) | Lognormal | GM = 3.1; GSD =2.79 | Nieuwenhuijsen et al. 1999 |
| C_air-T_ | Particulate matter concentration for transplanting (mg/m^3^) | Lognormal | GM = 0.96; GSD = 4.94 | Nieuwenhuijsen et al. 1999 |
| C_air-S_ | Particulate matter concentration for seeding (mg/m^3^) | Lognormal | GM = 0.96; GSD = 4.94 | Nieuwenhuijsen et al. 1999 |
| C_air-W_ | Particulate matter concentration for weeding (mg/m^3^) | Lognormal | GM = 5.11; GSD = 1.98 | Nieuwenhuijsen et al. 1999 |
| C_air-H mech_ | Particulate matter concentration for mechanized harvesting (mg/m^3^) | Lognormal | GM = 7.93; GSD = 3.13 | Nieuwenhuijsen et al. 1999 |
| C_air-H hand_ | Particulate matter concentration for hand harvesting (mg/m^3^) | Lognormal | GM = 2.02; GSD = 2.2 | Nieuwenhuijsen et al. 1999 |
| InhR | Inhalation rate, moderate intensity (m^3^/hour) | Normal | Mean = 1.8; SD = 0.49 | EPA EFH, Chapter 6 2011 |
| ED_BP_ | Exposure duration for bed preparation (hours) | Lognormal | GM = 99.1; GSD = 3.01 | Lupolt et al. 2024 |
| ED_S_ | Exposure duration for bed preparation (seeding) | Lognormal | GM = 34.2; GSD = 4.3 | Lupolt et al. 2024 |
| ED_T_ | Exposure duration for bed preparation (transplanting) | Lognormal | GM = 36.9; GSD = 4.3 | Lupolt et al. 2024 |
| ED_W_ | Exposure duration for weeding (hours) | Lognormal | GM = 85.5; GSD = 4.5 | Lupolt et al. 2024 |
| ED_H_ | Exposure duration for harvesting (hours) | Uniform | Min= 18.75; Max = 607.5 | Lupolt et al. 2024 |

**Table S2. Summary of the EPA RSL calculator selected reference doses and toxicity endpoint basis for PFOS, PFOA, PFNA, and PFDA**

| **PFAS** | RfD value (ng/kg/day) | EPA RSL calculator RfD source | Noncancer health endpoint basis, population |
| --- | --- | --- | --- |
| Perfluorooctanesulfonic acid (PFOS) | 0.1 | EPA Office of Water, 2024 | − Decreased birth weight, infants − Increased total cholesterol, adults |
| Perfluorooctanoic acid (PFOA) | 0.03 | EPA Office of Water, 2024 | − Decreased vaccine response to diphtheria and tetanus, children − Increased total cholesterol, adults |
| Perfluorononanoic acid (PFNA) | 3 | ATSDR, 2021 | − Decreased body weight and developmental delays, mice |
| Perfluorodecanoic acid (PFDA) | 0.002 | EPA IRIS, 2024 | − Decreased birth weight, infants − Decreased vaccine response to diphtheria and tetanus, children |

**Figure S1. Tornado plot for the ingestion soil screening level**

**Figure S2. Tornado plot for the dermal soil screening level**

**Figure S3. Tornado plot for the inhalation soil screening level**

**Figure S4. Tornado plot for the combined pathway soil screening level**

**Equation S1:** Combined soil screening level equation for farmworker

where:

**Supplemental information.** Cancer risk estimates for PFOS and PFOA

The incremental lifetime cancer risk was calculated by multiplying the reference dose (RfD) for PFOA and PFOS by their respective cancer slope factors (CSF) and adjusting the averaging time to lifetime (i.e., 70 years). As we are using a total hazard quotient (THQ) of 1 in our non-cancer equations, the exposure associated with the 5^th^ percentile SSL (which represents the 95^th^ percentile exposed persons) is equal to the RfD. Accordingly, the cancer risk estimates represent the risk for a 95^th^ percentile exposed person when soil levels are 26 ng/g for PFOS or 7.9 ng/g for PFOA.

|  | CSF^a^  (mg/kg/day)^-1^ | RfD (mg/kg/day) | Exposure duration (years) | Lifetime (years) | Cancer risk |
| --- | --- | --- | --- | --- | --- |
| PFOA | 2.93E+04 | 3.00E-08 | 26 | 70 | 3.3E-04 |
| PFOS | 3.95E+01 | 1.00E-07 | 26 | 70 | 1.5E-06 |

^a^ The CSF values were obtained from EPA RSL databases accessed on 12/2/2025: <https://www.epa.gov/risk/regional-screening-levels-rsls-generic-tables> .
